# Tumor-informed or tumor-agnostic circulating tumor DNA as a biomarker for risk of recurrence in resected colorectal cancer patients

**DOI:** 10.3389/fonc.2022.1055968

**Published:** 2023-01-26

**Authors:** Hiu Ting Chan, Satoshi Nagayama, Masumi Otaki, Yoon Ming Chin, Yosuke Fukunaga, Masashi Ueno, Yusuke Nakamura, Siew-Kee Low

**Affiliations:** ^1^ Project for Development of Liquid Biopsy Diagnosis, Cancer Precision Medicine Center, Japanese Foundation for Cancer Research, Tokyo, Japan; ^2^ Department of Gastroenterological and Surgery, Cancer Institute Hospital of the Japanese Foundation for Cancer Research, Tokyo, Japan; ^3^ Department of Surgery, Uji-Tokushukai Medical Center, Kyoto, Japan; ^4^ Department of Medical Oncology, Cancer Institute Hospital of the Japanese Foundation for Cancer Research, Tokyo, Japan; ^5^ Department of Clinical Chemotherapy, Cancer Chemotherapy Center, Japanese Foundation for Cancer Research, Tokyo, Japan; ^6^ Department of Research and Development, Cancer Precision Medicine, Inc., Kawasaki, Japan; ^7^ National Institutes of Biomedical Innovation, Health and Nutrition, Osaka, Japan

**Keywords:** circulating cell-free DNA, liquid biopsy, minimal residual disease, colorectal cancer, recurrence risk

## Abstract

**Introduction:**

Circulating tumor DNA (ctDNA) has been increasingly recognized as a promising minimally-invasive biomarker that could identify patients with minimal residual disease and a high risk of recurrence after definitive treatment. In this study, we’ve compared the clinical utility and sensitivity of 2 different approaches to ctDNA analyses: tumor-informed and tumor-agnostic in the management of colorectal (CRC) patients. The clinical benefits of a single timepoint ctDNA analysis compared to serial ctDNA monitoring after definitive treatment were also evaluated to uncover the ideal surveillance protocol.

**Methods:**

Patient-paired resected tumor tissues, peripheral blood cells, and a total of 127 pre-operative and serial plasma cell-free DNA (cfDNA) samples after definitive treatment from 38 CRC patients that had undergone curative intent surgery were analyzed using a commercial NGS cfDNA panel.

**Results:**

Up to 84% (32/38) of the recruited patients were detected with at least 1 genomic alteration from the tumor tissues that could be monitored using the tumor-informed ctDNA approach and none of the detected alterations were clonal hematopoiesis (CH) related. In contrast, 37% (14/38) of patients were detected with at least 1 monitoring alteration after exclusion of CH mutations using the tumor-agnostic approach. Serial plasma samples after definitive therapy were available for 31 patients. In the landmark ctDNA analysis, 24% (7/29) of patients had detectable ctDNA and were more likely to relapse than ctDNA-negative patients (p < 0.05). The landmark analysis sensitivity and specificity for recurrence were 67% and 87%, respectively. The incorporation of longitudinal ctDNA analysis at 6-months intervals improved the sensitivity to 100%. The median variant allele frequency (VAF) of the ctDNA mutations detected during surveillance was 0.028% (range: 0.018-0.783), where up to 80% (8/10) of the mutations were detected at VAF lower than the tumor-agnostic detection limit of 0.1%. Utilizing the tumor-agnostic approach reduced the recurrence detection sensitivity to 67% (4/6). Serial ctDNA analyses predicted disease recurrence at a median of 5 months ahead of radiological imaging.

**Conclusion:**

Longitudinal monitoring using tumor-informed ctDNA testing shows high analytical sensitivity, low probability of false-positive results due to CH mutations, and improved sensitivity in detecting recurrence which may modify the clinical management of CRC.

## Introduction

Colorectal cancer (CRC) is the third most common cancer in Japan leading to an estimated 51,000 deaths in 2019 (1). Despite improved surgical procedures and advances in treatment regimens, 30-40% of CRC patients develop recurrence within 5 years of post-definitive treatment (2, 3). The current standard of care for localized disease is surgery with complete mesocolic/mesorectal excision followed by adjuvant chemotherapy (ACT) in selected patients (4). Surgery-alone is currently recommended for all stage I patients with a 5-year-survival rate of over 90% (5). In contrast, ACT is recommended for high-risk stage II patients which are defined as those with poor prognostic features (T4 tumors, perforated tumors, bowel obstruction, perineural or lymphovascular invasion, poorly or undifferentiated tumor grade, grade BD3 tumor budding, and <12 lymph nodes removed) (2, 5, 6). However, the magnitude of survival benefits of adding ACT for high-risk stage II remains unclear and controversial, where a considerable number of patients have to suffer from the adverse effects of ACT without significant clinical benefit (7–9). For stage III CRC patients, up to 50% of patients can be cured from surgery alone and 20% of patients benefit from the additional ACT (10). Despite this, ACT is currently recommended for all stage III patients. Furthermore, up to 30% of the ACT-treated stage III patients would still develop recurrence, suggesting the need for additional therapy in this subset of patients (11). Effective clinical tools or biomarkers that identify patients who are more likely to recur after curative-intent therapy and may benefit from systemic treatments are greatly needed.

Circulating tumor DNA (ctDNA) has been increasingly recognized as a promising minimally-invasive biomarker that could detect minimal residual disease (MRD) in blood samples after definitive treatment and identify patients with a higher risk of recurrence (12–21). Several prospective interventional clinical trials are also underway to evaluate the clinical benefits of utilizing ctDNA for ACT guidance and detection of recurrence during disease surveillance (22–27). The majority of the conducted studies and clinical trials were designed based on tumor-informed ctDNA assays (12, 15–18, 20). A tumor-informed assay relies on initial genomic profiling of the tumor tissues to identify tumor-derived alterations that could be evaluated and monitored using ctDNA. This approach has shown high analytical sensitivity with an improved risk of recurrence prediction (28). However, recent studies have shown that tumor-agnostic assays, that are independent on prior tumor genomic knowledge of the patient, may also achieve comparable sensitivity to tumor-informed assay in identifying patients with a higher risk of recurrence (19, 21). Given the independence of tumor tissue sequencing, tumor-agnostic assays may offer a more rapid turnaround time with reduced cost. Nevertheless, limited studies have directly compared the clinical feasibility and sensitivity of both approaches.

The ongoing clinical trials have primarily focused on evaluating the clinical benefits of a single-time point ctDNA analysis (landmark ctDNA analysis) after definitive therapy for treatment guidance in CRC patients (22, 23). Based on the results from the current studies, it is evident that patients with detectable ctDNA at the landmark timepoint show a significantly inferior recurrence-free survival (RFS) compared to ctDNA-negative patients (12, 13, 17, 20). However, the results of these studies also indicated that 10-25% of the patients lacking detectable landmark ctDNA also recurred (12, 17, 19–21). These findings highlighted the potential inadequacy of a single timepoint ctDNA analysis to predict recurrence and guide treatment decisions. The integration of longitudinal and surveillance ctDNA analysis may improve the prediction of recurrence risk (16, 19, 20), however, the most optimal approach and surveillance protocol for identifying high-risk CRC patients remain unclear.

In this study, we report findings from a prospective and observational study that compared the clinical feasibility and sensitivity of utilizing a commercially available cfDNA panel with a tumor-informed, and tumor-agnostic approach to predict the risk of recurrence in the same resected CRC patient cohort. Plasma ctDNA analysis was performed before surgical resection and during routine follow-up after curative-intent treatment to assess the clinical utility of both landmark and longitudinal ctDNA monitoring in predicting the risk of recurrence.

## Materials and methods

### Patient cohort and sample collection

**Figure 1:**
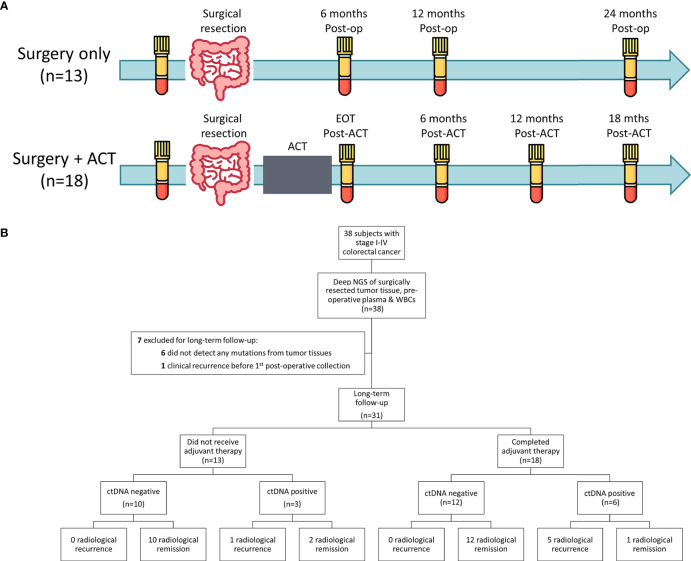
Study design and patient enrolment. **(A)** Study design and overview of the blood collection time points. For patients treated with surgery alone (n=13), blood samples were collected before surgical resection and at 6 months, 12 months and 24 months after surgery (Post-op) or until radiological recurrence. For patients who received adjuvant chemotherapy (ACT), blood samples were collect prior to surgical resection, at the end of ACT (EOT) and at 6 months, 12 months and 18 months after completion of ACT (Post-ACT) or until radiological recurrence. **(B)** A total of 38 stage I-IV CRC patients that undergone surgical resection with curative-intent were included in this study. After exclusion, 31 patients were included for long-term follow-up analysis. Circulating tumor DNA status was determined based on the longitudinal tumor-informed ctDNA analysis after definitive treatment.

A total of 38 patients with histologically confirmed colorectal adenocarcinoma from the Cancer Institute Hospital of Japanese Foundation for Cancer Research in 2018 were included in this study. All eligible patients included in this study were pathologically confirmed as stage I to IV colorectal adenocarcinoma and were not subjected to chemotherapy or radiation therapy before tumor resection. The clinical and pathological information was obtained from the pathology reports and the electronic medical record for each patient. This study was approved by the ethical committee in Japanese Foundation for Cancer Research (IRB-2013-1093). The study design and details of blood collection time points are shown in **Figure 1A**. Tumor tissues and peripheral blood samples were collected at the time of surgery. Blood samples were collected longitudinally after surgical resection and completion of adjuvant chemotherapy and evaluated retrospectively. For patients who only underwent surgical resection, monitoring blood samples were collected at 6 months, 12 months, and 24 months after surgery. For patients administered ACT, blood samples were collected at 0, 6, 12, and 18 months after completion of ACT. Surgically-resected tumor tissues were stored at -80 ˚C until DNA extraction. The collection and processing of blood have been described previously (29–32). Briefly, 14 mL of peripheral blood was collected using EDTA-2Na tubes (Terumo, Tokyo, Japan) and were centrifuged at 2,000x *g* at 4 ˚C for 10 minutes within 30 minutes after the collection. The obtained plasma samples were further centrifuged at 16,000x *g* at 4 ˚C for 10 minutes to remove cell debris. The separated plasma and peripheral blood cells (PBCs) were stored at -80 ˚C until nucleic acid extraction.

### DNA/RNA extraction

A total of 127 pre-and post-operative plasma samples were collected from 38 patients and the cell-free total nucleic acid (cfTNA), which includes both DNA and RNA, was extracted using the MagMAX Cell-Free Total Nucleic Acid Isolation kit (Applied Biosystems) according to the manufacturer’s protocol. Genomic DNA was extracted from frozen tumor tissues using the Allprep DNA Mini Kit (Qiagen) according to the manufacturer’s protocol. Frozen PBCs samples were treated with the Red Blood Cell Lysis buffer following the manufacturer’s protocol (BioLegend). The treated PBCs were counted using the Invitrogen Countess Automated Cell counter (Fisher Scientific) and DNA from a total of 2 × 10^6^ PBCs was extracted using the Allprep DNA Mini Kit. Extracted cfTNA and genomic DNA (both PBCs and tumor tissues) were quantified using Qubit DNA HS Assay Kit and Qubit DNA Broad range assay kit (Life Technologies), respectively. The quality of the extracted DNA was assessed using the TapeStation system (Agilent) either *via* Genomic DNA ScreenTape (tumor and PBCs DNA) or High Sensitivity D5000 ScreenTape (cfTNA) (Agilent).

### Library preparation and targeted next-generation sequencing

Targeted NGS for cfTNA was carried out using the Oncomine Pan-Cancer Cell-Free Assay following the manufacturer’s protocol (Life Technologies), with an input of 8.3-20 ng of cfTNA. Oncomine Pan-Cancer Cell-Free Assay is an amplicon-based ctDNA targeted assay with unique molecular identifiers (UMIs) and detects single nucleotide variants (SNVs), copy number variations (CNVs), and gene arrangements across 52 genes. Library construction was undertaken as previously described (29–32). Libraries were multiplexed for templating on the Ion Chef Instrument and subsequently sequenced on the Ion S5 Prime System using the Ion 540 or 550 Chip Kit. Both tumor and PBCs DNA were mechanically sheared to 150 bps before library construction. A similar sequencing methodology was applied for DNA extracted from tumor tissue and PBCs with an input of 20 ng.

### Sequencing data analysis and statistical analysis

Sequencing alignment, quality control analysis, and variant calling were conducted by the Torrent Suite Software version 5.10.1 (Thermo Fisher Scientific) and Ion Reporter version 5.10 (Thermo Fisher Scientific). In brief, raw sequence files were aligned to hg19 using the Torrent Mapping Alignment Program (TMAP) with default analysis parameters. The subsequent BAM files generated were then further analyzed by Oncomine TagSeq Pan-Cancer Liquid Biopsy w2.1 version 5.10 with the following modifications for a positive variant calling: (i) A minimum of 3 reads with the same UMI were required to form a functional family. (ii) Under tumor-agnostic calling, a minimum of 3 variant supporting functional families with a minimum variant allele frequency (VAF) of 0.1% were required to make single nucleotide variants (SNVs), multi nucleotide variants (MNVs), and insertions/deletions (INDELs) callings for a known cancer hotspot mutation. (iii) Under the matched tumor-informed manner where the mutation was previously detected from the tumor tissue of a patient, a minimum of 1 variant supporting functional family was required to make SNVs, MNVs, and INDELs callings. Variants were annotated using Oncomine Pan-Cancer Annotation version 1, a proprietary list of databases. RFS was assessed by standard radiologic criteria. RFS was measured from the day of completion of definitive treatment to the first verified radiological recurrence. For patients whose treatment was only surgery, RFS was measured from the day of surgical resection. For patients who received adjuvant chemotherapy, RFS was measured from the day of completion of chemotherapy. The definition of RFS was similarly described in a previous study (19). Patients were censored at the date of the last follow-up. Survival analysis was performed using the Kaplan-Meier method. Cox proportional hazards regression analysis was used to assess the association of ctDNA with RFS. Differences in pre-operative ctDNA detection rate between tumor-informed and tumor-agnostic approaches and differences in recurrence rate between ctDNA positive and ctDNA negative groups were assessed using Fisher’s exact test. All p-values were based on two-sided testing and differences were considered significant at p<0.05. Statistical analysis was performed using R Statistical software (Version 4.0.5).

## Results

### Patient characteristics

An overview of the study workflow is presented in **Figure 1B**. A total of 38 CRC patients were included in this study. The clinical and pathological characteristics of the patients are shown in **Table 1** and **Table S1**. The median age of the patients at the initial sample collection was 66 years old and 63% of the patients were male. Among them, 53% (20/38) were diagnosed with stage I or II, and 47% (18/38) were diagnosed with stage III or IV. All patients underwent surgical resection with curative intent. One of the three Stage IV patients received simultaneous resection of the primary tumor and solitary liver metastatic lesion, and the remaining two Stage IV patients underwent the resection of the solitary peritoneal dissemination along with the primary tumor. Six patients with no genomic alterations detected from tumor tissues and one patient who developed clinical recurrence before the collection of the first post-operative blood sample were excluded for long-term follow-up (**Figure 1B**).

**Table 1:** Clinical and pathological characteristics of the study cohort.

Clinico-pathologic features	No., (%) (n=38)
Age, years	
Median	66
Range	42-88
Gender	
Male	24 (63)
Female	14 (37)
Stage	
I	7 (18)
II	13 (34)
III	15 (40)
IV	3 (8)
Tumor Site	
Cecum	6 (16)
Ascending	4 (11)
Transverse	4 (11)
Descending	2 (5)
Sigmoid	5 (13)
Rectum	17 (45)
Differentiation	
Well	8 (21)
Moderate	28 (74)
Poor	2 (5)
T Stage	
T1	4 (11)
T2	4 (11)
T3	20 (53)
T4	10 (26)
Nodal involvement	
N0	21 (55)
N1,N2,N3	17 (45)
Tumor size (mm)	
Median	40
Range	12-90
Lymphatic Invasion	
No	20 (53)
Yes	18 (47)
Venous Invasion	
No	9 (24)
Yes	29 (76)
Baseline CEA elevated (>5 ng/mL)	
No	26 (68)
Yes	12 (32)
Baseline CA 19-9 elevated, (>37 U/mL)	
No	35 (92)
Yes	3 (9)
Baseline CA-125 elevated,(>46 U/mL)	
No	38 (100)
Yes	0 (0)

ACT was administered to 18/31 longitudinally monitored patients with a median chemotherapy duration of 179 days (**Table S2**). Radiological recurrence was detected in 19% (6/31) of the evaluated patients with a median time to recurrence of 8.5 months after definitive treatment. The median follow-up time was 20 months (14-27 months) after definitive treatment for recurrence-free patients.

### Detection of pre-operative ctDNA using the tumor-agnostic or tumor-informed approach

Cell-free TNA was successfully extracted from all 127 plasma samples with an average concentration of 8.0 ng per mL of plasma (1.6-36.9 ng/mL; **Table S3**). All cfTNA samples were successfully sequenced with an average cell-free DNA (cfDNA) input of 17.5ng to a median raw coverage of 54,772x and a median collapsed coverage of 4,296 (**Table S3**). The average library conversion rate was 82% (**Table S4**). Under a tumor-agnostic setting with a VAF detection limit of 0.1%, a total of 27 SNVs and Indels were detected in the pre-operative plasma samples from 50% (19/38) of the patients with a median VAF of 0.37% (Range: 0.10-14.25%; **Table S5**). Genomic DNA extracted from pre-operative PBCs was sequenced to a comparable coverage to the plasma cfDNA (raw coverage of 48,622x and median collapsed coverage of 2,581x; **Table S6**) to identify the possible clonal-hematopoiesis (CH) mutations. A total of 11 mutations were detected from PBCs and 9 of them were simultaneously detected from plasma cfDNA (**Table S7**). CH mutations constituted up to 33.3% (9/27) of the total mutations detected from plasma cfDNA (**Table S5**). After the exclusion of CH mutations, 37% (14/38) of the evaluated patients harbor at least one tumor-derived mutation from plasma cfDNA for longitudinal monitoring.

**Figure 2:**
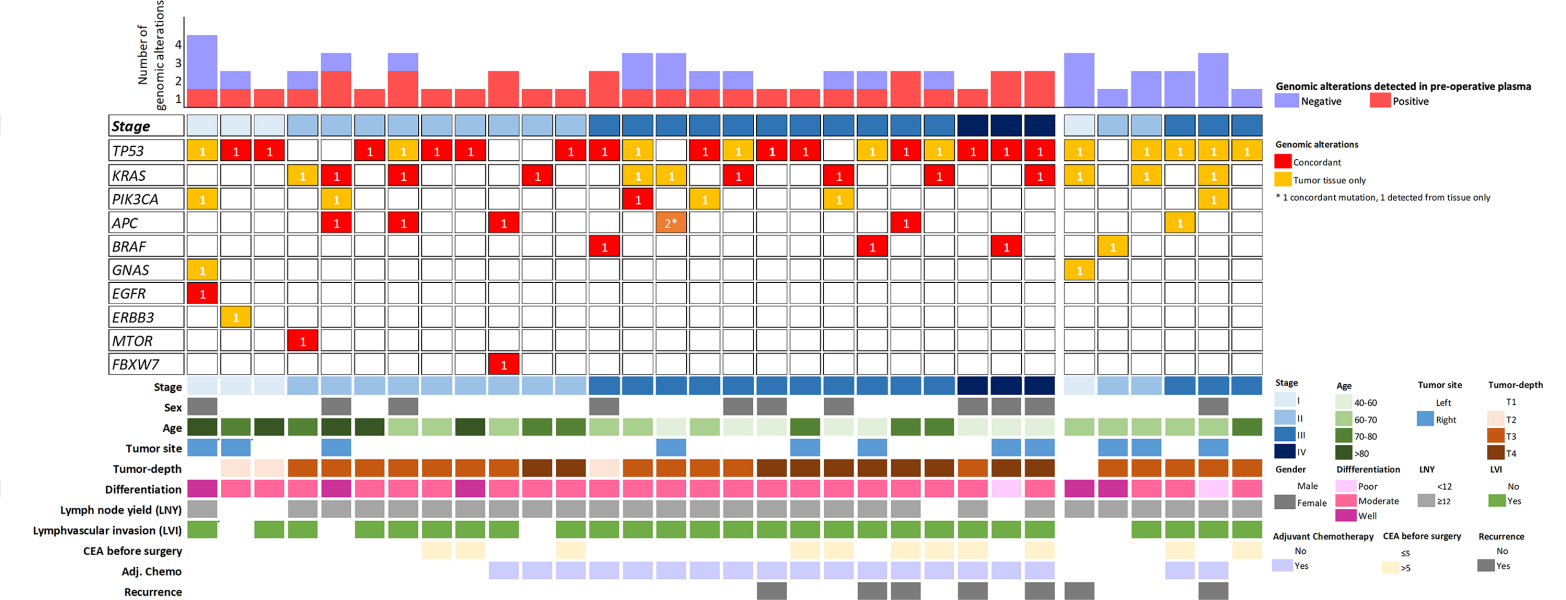
A schematic representation of the genomic alteration distribution detected from tumor tissues and pre-operative plasma cfDNA. Each column represents a sample, and it is classified according to the pathological stage. Six patients with no detectable ctDNA were group on the right and six patients with no genomic alterations detected from the tumor tissues were excluded from the plot.

**Figure 3:**
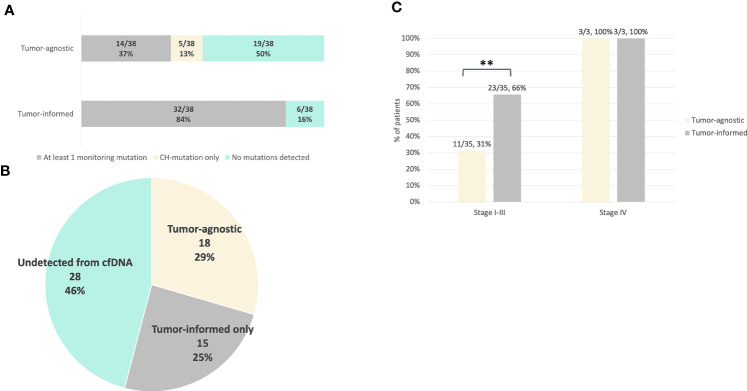
Comparison of pre-operative ctDNA detection between the tumor-informed and tumor-agnostic approach. For tumor-informed approach, mutation detection was evaluated based on the genomic profile of the tumor tissues. For tumor-agnostic evaluation, mutation detection was evaluated based on alterations detected from pre-operative plasma cfDNA with VAF ≥ 0.1% without previous knowledge of the tumor genomic profile (Methods). Clonal hematopoiesis (CH) mutations were excluded from both approaches. **(A)** Number of patients detected with at least one monitoring tumor-derived mutation for ctDNA surveillance using the tumor-agnostic or tumor-informed approach. **(B)** Proportion of tumor mutations that were detected from pre-operative plasma cfDNA using the tumor-agnostic or tumor-informed approach. A total of 61 mutations were detected from tumor tissues, 18/61 were detected from plasma cfDNA using the tumor-agnostic approach and additional 15 alterations were detected from pre-operative plasma cfDNA using the tumor-informed assay. **(C)** Pre-operative ctDNA detection rates in stage I-III (n=35) and stage IV (n=3) patients using tumor-informed and tumor-agnostic ctDNA testing (p value = 0.00806). The p value was obtained from two-by-two Fisher’s exact tests. ** p < 0.01.

The genomic profile of tumor tissues was evaluated to compare the detection of pre-operative ctDNA between tumor-agnostic and tumor-informed approaches. Genomic DNA isolated from tumor tissues was sequenced to a median coverage of 18,450x (**Table S8**). A total of 61 somatic genomic alterations in 10 genes were identified using a VAF cut-off of 1% (**Table S9**). The mutational landscape of the detected mutations is summarized in **Figure 2**, where the most commonly mutated genes were *TP53* (41%), *KRAS* (21%), *PIK3CA* (11%), and *APC* (10%) (**Supplementary Figure 1**). Using a VAF cut-off of 1%, none of the mutations detected from tumor tissues were present in the PBCs (**Table S10**). In contrast to the tumor-agnostic approach, up to 84% (32/38) of the patients harbor at least one mutation from the tumor tissue for subsequent plasma cfDNA monitoring (**Figure 3A**). Of those mutation calls from tumor tissues, 29.5% (18/61) were concordantly detected from pre-operative plasma without the aid of prior patient-specific tumor genomic knowledge (**Figure 3B**). An additional 24.6% (15/61) of the alterations from tumor tissues were detected from plasma cfDNA using the tumor-informed approach, with a minimum of 1 variant supporting functional family (Method) and a median observed VAF of 0.04% (Range: 0.02-0.09% **Figure 3B**). The pre-operative ctDNA detection rate was significantly higher in stage I-III CRC patients using the tumor-informed approach compared to the tumor-agnostic approach with a detection rate of 66% and 31% respectively (p-value = 0.008; **Figure 3C** and **Supplementary Figure 2**). In contrast, ctDNA was detected in all three stage IV patients using both approaches. Due to the higher detection sensitivity observed using the tumor-informed approach, all subsequent post-therapy cfDNA samples were analyzed using the tumor-informed approach.

### Landmark ctDNA analysis after definitive treatment and risk of recurrence

**Figure 4:**
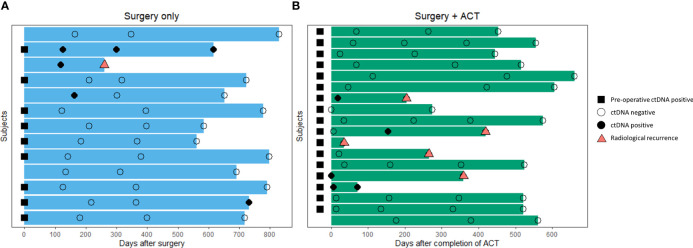
Overview of blood samples analyzed for landmark and longitudinal ctDNA testing after definitive therapy. **(A)** Patients that received curative-intent surgery alone (n=13) **(B)** Patients that received curative-intent surgery followed by adjuvant chemotherapy (n=18).

**Figure 5:**
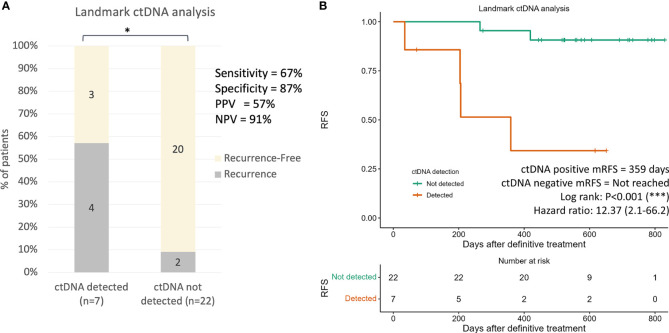
Landmark ctDNA analysis for recurrence risk assessment. **(A)** Recurrence rates in patients with detected ctDNA and undetected ctDNA at landmark ctDNA analysis (*p* value=0.01080). The *p* value was obtained from two-by-two Fisher’s exact tests. **(B)** Kaplan-Meier plot of recurrence-free survival stratified for ctDNA detection in landmark ctDNA analysis. * p < 0.001; *** p < 0.0001.

‘Landmark’ ctDNA analysis was defined as the detection of ctDNA from the first plasma sample drawn after the completion of definitive treatment (surgery alone or completion of adjuvant chemotherapy). For patients who were subjected to surgery alone, the landmark sample was taken approximately 5 months after surgery (median: 162 days, **Figure 4A**). For patients who have received adjuvant chemotherapy, the first plasma sample was taken approximately 1 month after completion of adjuvant chemotherapy (median 22.5 days, **Figure 4B**). Landmark plasma samples were available for 29 of the 31 patients with long-term follow-up, and ctDNA was detected in 24% (7/29) of samples (**Figure 5A**). The recurrence rate was significantly higher for ctDNA-positive patients at 57% (4/7), compared to 9% (2/22) for negative patients (p<0.05, **Figure 5A**). Sensitivity and specificity for detection of recurrence were 67% and 87% respectively (**Figure 5A**). Recurrence-free survival for patients with detectable landmark ctDNA was significantly shorter than those with negative ctDNA and a 12.4 times higher risk of developing recurrence (**Figure 5B**, HR: 12.4, P<0.001).

### Longitudinal ctDNA and risk of recurrence

**Figure 6:**
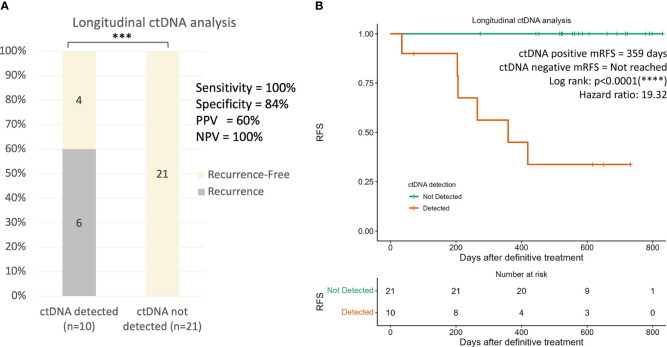
Longitudinal ctDNA analysis for recurrence risk assessment. **(A)** Recurrence rates in patients with detected ctDNA and undetected ctDNA at longitudinal ctDNA analysis (*p* value=0.002852). Detection of ctDNA at any serial plasma samples until the development of clinical recurrence would be considered as ctDNA-positive. The *p* value was obtained from two-by-two Fisher’s exact tests. **(B)** Kaplan-Meier plot of recurrence-free survival stratified for ctDNA detection in longitudinal ctDNA analysis. ***p < 0.05; ****p < 0.001.

To investigate whether longitudinal ctDNA analyses could improve the sensitivity for recurrence prediction compared to landmark analysis, subsequent plasma samples were evaluated for all 31 patients with long-term follow-up. A total of 3 or 4 serial plasma samples after the end of definitive treatment (surgery only or ACT, respectively) from each patient were drawn for the longitudinal ctDNA analysis (**Table S10**). Detection of ctDNA at any serial plasma samples until the development of clinical recurrence would be considered ctDNA-positive. Overall, 60% (6/10) of patients who were tested ctDNA-positive during surveillance developed radiological recurrence, whereas none of the 21 patients that remained ctDNA-negative throughout the surveillance developed clinical recurrence, giving a negative predictive value of 100% (**Figure 6A**, p<0.001). The incorporation of serial ctDNA increased the sensitivity of prediction for recurrence from 67% to 100% (**Figure 6A**), and ctDNA-positive patients remained to have a significantly lower RFS compared to negative patients (HR:19.3, p<0.0001, **Figure 6B**). The median VAF of the detected mutations was 0.028% (range: 0.018-0.783), and up to 80% (8/10) of the mutations were detected at VAF lower than the tumor-agnostic detection limit of 0.1% (**Supplementary Figure 3A**). Consequently, only 67% (4/6) of the recurrence cases could be detected using tumor-agnostic ctDNA monitoring (**Supplementary Figures 3B, C**).

**Figure 7:**
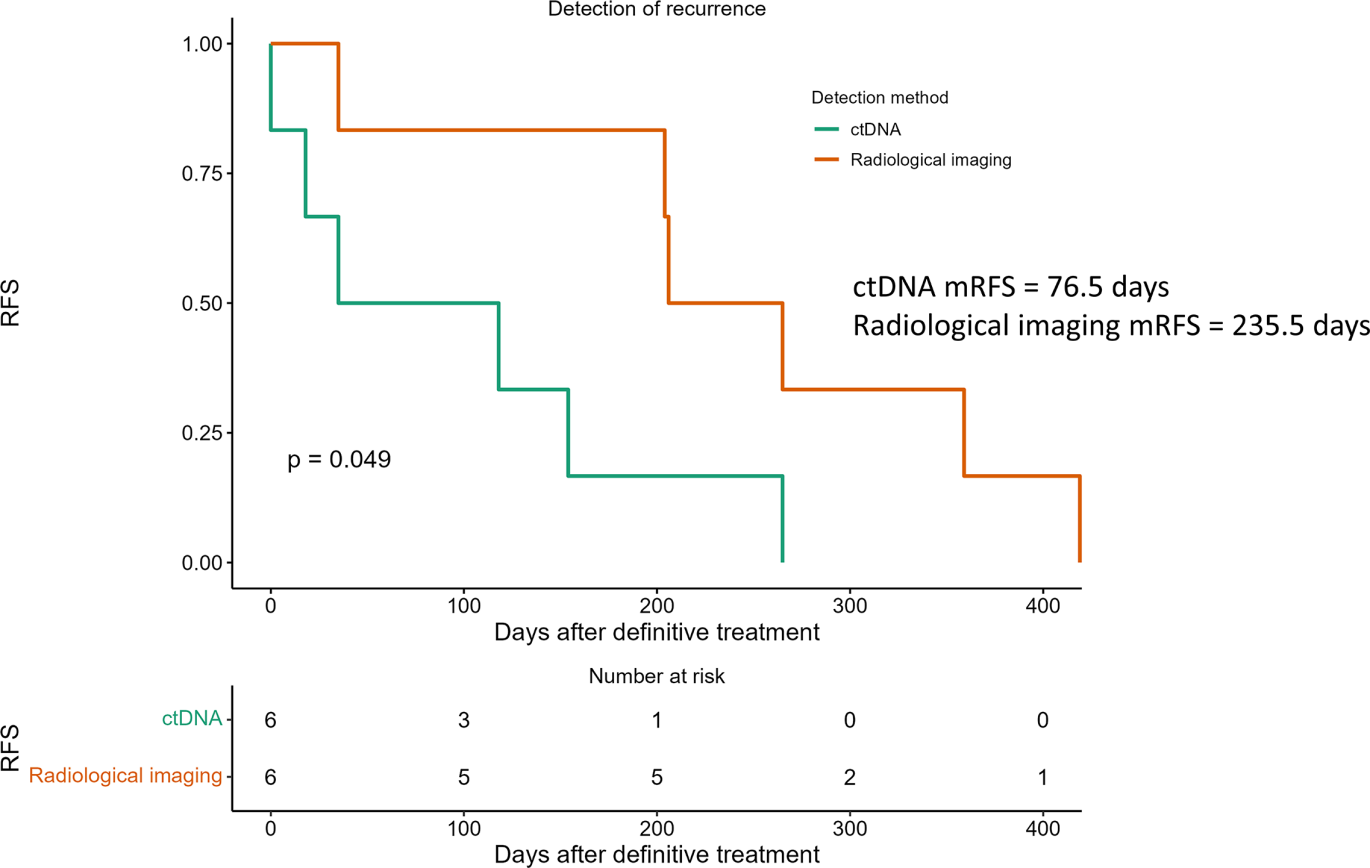
Detection of recurrence using longitudinal ctDNA analysis and standard radiological imaging. ctDNA (green line) analyzed at serial time points after definitive treatment predicted recurrence with a median lead time of 159 days over radiological recurrences (red line). P value determined by log-rank test.

Longitudinal ctDNA analysis detected recurrence earlier than radiological imaging in 4 of the 6 recurrence patients (**Supplementary Figure 4**). The median time for disease recurrence determined by ctDNA analyses was 77 days after definitive treatment, compared to 236 days determined by radiological imaging, resulting in a ctDNA median lead time of 159 days (**Figure 7**, p<0.05). In the 3 recurrence cases where plasma samples were available at the time of radiological detection of relapse, ctDNA remained positive with an increase in the VAF of the detected mutations in all patients at the time of radiological recurrence (mean 10.1 folds, range: 2.9-16.8 folds), indicating the markedly increase in tumor burden while the patients awaited radiologic detection of the recurrence (**Supplementary Figure 5**).

## Discussion

A sensitive prognostic biomarker that accurately identifies CRC patients with a higher risk of recurrence after curative-intent therapy could potentially improve their survival outcome. The results from our observational study are in concordance with previously published studies where ctDNA analysis after definitive treatment has demonstrated significant therapeutic promise in identifying patients with poor prognoses who may require further systemic treatments. Our study also emphasizes the importance of using a tumor-informed ctDNA assay with longitudinal surveillance to optimize the clinical utility of ctDNA in CRC management.

In the pre-operative context, we’ve observed that the tumor-informed ctDNA approach may offer superiority in detecting low tumor burden, especially in localized CRC patients over the tumor-agnostic approach. Up to 84% of our patient cohort was detected with at least one mutation from the tumor tissues that could be subsequently monitored using ctDNA, compared to only 37% of patients detected with at least one monitoring mutation using the tumor-agnostic approach. Without prior knowledge of the tumor genomic profile of the patient, the reason for the absence of ctDNA detection in the remaining 63% of patients would remain unknown as to whether it is due to the insufficient coverage of the targeted panel or low tumor fraction in the cfDNA, resulting further ctDNA monitoring for these patients to be clinically nonmeaningful. The significantly higher pre-operative ctDNA detection rate observed in stage I-III patients using a tumor-informed approach compared to the tumor-agnostic approach (66% and 31%, respectively) further illustrates the loss of sensitivity associated with the tumor-agnostic approach. The enhanced ctDNA detection sensitivity using a tumor-informed manner has been similarly demonstrated using the MSK-ACCESS ctDNA assay (33). The authors of the study reported that by performing variant calling in a matched tumor-informed manner, an additional 5% of variants were detected from the plasma cfDNA (33). Interestingly, ctDNA was detected in all three metastatic patients from our study cohort using both the tumor-informed and tumor-agnostic approach, highlighting that the impact of assay sensitivity is more prominent in patients with localized CRC. Furthermore, 33% of the alterations detected from pre-operative plasma cfDNA were of CH origin and the detection frequency was consistent with previous studies (34–37). We’ve previously reported that the type and VAF of CH alterations detected from plasma cfDNA are often indifferent to ctDNA mutations, therefore patient-paired PBCs sequencing is essential to differentiate CH from tumor-derived alterations (29, 38). Misclassification of the origin of the alterations detected from cfDNA may lead to an erroneous interpretation of ctDNA analysis as an MRD (29). In contrast, using a VAF threshold of 1%, none of the patients in our study cohort were detected with CH alterations from the tumor tissues, precluding the need for additional sequencing of PBCs when adopting the tumor-informed approach.

In the post-definitive therapy context, 32% of our patient cohort was detected with ctDNA in at least one of the surveillance plasma samples yielding a recurrence detection sensitivity of 100%. Our observation aligns with previously reported tumor-informed ctDNA assays that produced sensitives ranging from 80-100% (15–18, 20). Up to 80% of the variants identified from the first ctDNA positive monitoring samples were detected at VAF below the tumor-agnostic detection limit (VAF<0.1%). Consequently, only 67% (4/6) of the recurrence cases could be detected using tumor-agnostic ctDNA monitoring. Similar detection sensitivity has been reported in a recent study that evaluated the feasibility of tumor-uninformed MRD detection using a plasma-only ctDNA assay with 73% of recurrence patients detected using ctDNA surveillance (19). Moreover, the authors also observed an increase in sensitivity by 18% with the incorporation of aberrant methylation patterns (19). Several other studies have also explored the use of epigenomic features in tumor-agnostic ctDNA assays for MRD detection. The reported sensitivity in detecting recurrence ranged from 63-90% (21, 39, 40). Current observations suggest genomic alterations-based tumor-agnostic ctDNA assays are unlikely to achieve comparable sensitivity as the tumor-informed approach in detecting and predicting recurrence in resectable CRC patients. The incorporation of other features is necessary for tumor-agnostic ctDNA assays to be used in clinical settings. Although the tumor-informed ctDNA approach outperforms the tumor-agnostic assay in terms of analytical and clinical sensitivity, the clinical utility of a tumor-informed ctDNA assay will inevitably be significantly reduced in cases where tumor tissues are not available or with limited tumor cellularity. This issue may be particularly relevant in patients that have undergone neoadjuvant therapy where resected specimens may have insufficient tissue or tumor content for genomic profiling due to following favorable treatment response.

Landmark ctDNA analysis after completion of definitive treatments is clinically attractive as it may facilitate immediate decision-making for initiation of adjuvant treatments or consolidation therapies. Consistent with previous studies, patients from our study cohort detected with positive ctDNA at the landmark sample showed an inferior RFS and 12 times higher risk of developing recurrence compared to ctDNA negative patients (12–21). Together, these results have suggested the possibility of treatment escalation in ctDNA-positive patients and treatment de-escalation in ctDNA-negative patients. The clinical benefits of the ctDNA-guided treatment approach in stage II CRC patients were recently reported for the first time from a phase II randomized prospective and interventional trial where the ctDNA-guided approach was able to reduce ACT usage in stage II patients without compromising RFS compared to the standard management (24). However, insufficient sensitivity of single timepoint analyses resulting in false-negative results may undermine the ctDNA-guided treatment regimen. Previous studies together with the aforementioned interventional trial have shown that close to 10% of patients with undetectable ctDNA after definitive treatment develop recurrence (12, 17, 24). This was similarly observed in our patient cohort where landmark ctDNA analysis was able to detect 67% of the recurrence cases with a relapse rate of 9% among the ctDNA negative patients. Future studies should explore the incorporation of ctDNA analysis with other circulating analytes such as circulating tumor cells, and non-genomic features to improve the sensitivity of landmark analysis in identifying patients with a higher risk of recurrence (41). One other strategy to alleviate the sensitivity-related issue is through longitudinal ctDNA testing. Previous studies have reported an increase in sensitivity from 40% to 88% using serial ctDNA analyses (17, 20). Similarly, we’ve also observed an increase in sensitivity from 67% to 100% through six-monthly ctDNA testing compared to a single timepoint analysis. These data highlight the importance of incorporating longitudinal ctDNA monitoring to maximize the clinical benefits of ctDNA analyses. Surveillance of ctDNA after definitive treatment showed a significant impact in recurrence detection compared to radiological imaging, demonstrating a lead time of 5 months that is similar to previously reported (12, 17, 20). In the cases where ctDNA analyses were also performed at the time of radiological recurrence, the ctDNA levels increased by a mean of 10 folds, indicating a marked increase of tumor burden during the 5 months of lead time. The early detection of residual disease from ctDNA analyses may allow earlier radiological imaging to be performed or timely adjustment of the treatment regimens. Some of the ongoing interventional trials have adopted the ctDNA surveillance approach in the study design where radiological imaging frequencies and ACT dosage regimen are modified according to the ctDNA status every 3 to 4 months (26, 27).

There are several limitations to our study. The small sample size and the low event rate limited our ability to compare the prognostic significance of ctDNA status with other known clinical features. Future studies with a larger cohort size are needed to validate this. The specificity of longitudinal ctDNA analysis observed in our cohort was lower than reported in previous observational studies (84% and 95%, respectively) (17, 20). One of the four ctDNA-positive patients was lost in follow-up, while one patient with detected ctDNA at the end of the monitoring period was diagnosed with intrahepatic cholangiocarcinoma. Further evaluations for this patient are needed to confirm the origin of the radiologically detected tumor and to assess the discrepancies between ctDNA analysis and the clinical diagnosis. The remaining two patients were monitored for approximately 16 months after the first detection of ctDNA from the surveillance samples. In the study conducted by Henriksen et al., the authors observed 2 distinct tumor growth patterns where half of the recurrence patients showed slow growth with longer overall survival (20). It is unclear whether the slow growth pattern may account for the 2 ctDNA positive patients that remained undetected using radiological imaging by the end of the monitoring period. In this study, we’ve utilized a commercially available targeted cfDNA panel instead of establishing a personalized cfDNA assay based on the patient’s tumor tissue genomic profile. Using a generic assay shortens the turnaround time and reduces the cost, however, up to 16% of the recruited patients from the study were excluded for further monitoring due to the lack of alterations detected from tumor tissues. The limited panel coverage for genes *APC* and *TP53*, and the use of a hot-spot-based variant calling bioinformatic pipeline with limited *de novo* calling may account for the reduced coverage observed. Improvements to the variant calling algorithm may overcome this drawback.

## Conclusion

In summary, we showed that ctDNA analysis with the tumor-informed approach outperforms the tumor-agnostic approach with higher analytical sensitivity, lower probability of false-positive results due to CH mutations, and improved sensitivity in detecting recurrence in resected CRC patients. Our results have also demonstrated that serial ctDNA monitoring after definitive treatment provides superior sensitivity over landmark ctDNA analyses in predicting and detecting recurrence. These data also suggest the clinical importance of incorporating longitudinal ctDNA monitoring to maximize the clinical benefits of liquid biopsy in CRC management.

## Data availability statement

The original contributions presented in the study are publicly available. This data has been uploaded to the NBDC database with the accession number JGAS000590.

## Ethics statement

The studies involving human participants were reviewed and approved by Ethical committee in Japanese Foundation for Cancer Research (IRB-2013-1093). The patients/participants provided their written informed consent to participate in this study.

## Author contributions

The study concept and design were conducted by SN, YN, and S-KL. Patient recruitment was carried out by SN, YF, and MU. Acquisition, analysis, or interpretation of data were performed by HC, SN, YC, and S-KL. Drafting of the manuscript was written by HC, SN, and S-KL. HC, SN, YC, YN, and S-KL contributed critical revision of the manuscript for important intellectual content. HC, MO provided technical support. SN, YF, MU, YN, and S-KL were the supervisors for this project. All authors have read and approved the final manuscript.

## References

[1] Foundation for Promotion of Cancer Research. Cancer statistics in Japan- 2021. (2021). https://ganjoho.jp/public/qa_links/report/statistics/2021_en.html

[2] Brenner H Kloor M Pox CP. Colorectal cancer. Lancet (9927) 2014:1490–502:383. doi: 10.1016/S0140-6736(13)61649-9 24225001

[3] Buccafusca G Proserpio I Tralongo AC Rametta Giuliano S Tralongo P. Early colorectal cancer: Diagnosis, treatment and survivorship care. Crit Rev Oncol Hematol (2019) 136:20–30. doi: 10.1016/j.critrevonc.2019.01.023 30878125

[4] Dienstmann R Salazar R Tabernero J. Personalizing colon cancer adjuvant therapy: selecting optimal treatments for individual patients. J Clin Oncol (2015) 33(16):1787–96. doi: 10.1200/JCO.2014.60.0213 25918287

[5] Hashiguchi Y Muro K Saito Y Ito Y Ajioka Y Hamaguchi T. Japanese Society for cancer of the colon and rectum (JSCCR) guidelines 2019 for the treatment of colorectal cancer. Int J Clin Oncol (2020) 25(1):1–42. doi: 10.1007/s10147-019-01485-z 31203527 PMC6946738

[6] Baxter NN Kennedy EB Bergsland E Berlin J George TJ Gill S. Adjuvant therapy for stage II colon cancer: ASCO guideline update. J Clin Oncol (2022) 40(8):892–910. doi: 10.1200/JCO.21.02538 34936379

[7] Rebuzzi SE Pesola G Martelli V Sobrero AF. Adjuvant chemotherapy for stage II colon cancer. Cancers (Basel) (2020) 12(9):2584. doi: 10.3390/cancers12092584 32927771 PMC7565376

[8] Quasar Collaborative G Gray R Barnwell J McConkey C Hills RK Williams NS. Adjuvant chemotherapy versus observation in patients with colorectal cancer: a randomised study. Lancet (2007) 370(9604):2020–9. doi: 10.1016/S0140-6736(07)61866-2 18083404

[9] O'Connor ES Greenblatt DY LoConte NK Gangnon RE Liou JI Heise CP. Adjuvant chemotherapy for stage II colon cancer with poor prognostic features. J Clin Oncol (2011) 29(25):3381–8. doi: 10.1200/JCO.2010.34.3426 PMC3164243 21788561

[10] Taieb J Andre T Auclin E. Refining adjuvant therapy for non-metastatic colon cancer, new standards and perspectives. Cancer Treat Rev (2019) 75:1–11. doi: 10.1016/j.ctrv.2019.02.002 30849607

[11] Smoragiewicz M Lim H Peixoto RD. Surveillance for asymptomatic recurrence in resected stage III colon cancer: does it result in a more favorable outcome? J Gastrointest Oncol (2015) 6(3):268–73. doi: 10.3978/j.issn.2078-6891.2015.019 PMC4397253 26029453

[12] Tie J Wang Y Tomasetti C Li L Springer S Kinde I. Circulating tumor DNA analysis detects minimal residual disease and predicts recurrence in patients with stage II colon cancer. Sci Transl Med (2016) 8(346):346ra92. doi: 10.1126/scitranslmed.aaf6219 PMC5346159 27384348

[13] Tie J Cohen JD Wang Y Christie M Simons K Lee M. Circulating tumor DNA analyses as markers of recurrence risk and benefit of adjuvant therapy for stage III colon cancer. JAMA Oncol (2019) 5(12):1710–7. doi: 10.1001/jamaoncol.2019.3616 PMC6802034 31621801

[14] Tie J Wang Y Cohen J Li L Hong W Christie M. Circulating tumor DNA dynamics and recurrence risk in patients undergoing curative intent resection of colorectal cancer liver metastases: A prospective cohort study. PLoS Med (2021) 18(5):e1003620. doi: 10.1371/journal.pmed.1003620 33939694 PMC8128260

[15] Wang Y Li L Cohen JD Kinde I Ptak J Popoli M. Prognostic potential of circulating tumor DNA measurement in postoperative surveillance of nonmetastatic colorectal cancer. JAMA Oncol (2019) 5(8):1118–23. doi: 10.1001/jamaoncol.2019.0512 PMC6512291 31070668

[16] Tarazona N Gimeno-Valiente F Gambardella V Zuniga S Rentero-Garrido P Huerta M. Targeted next-generation sequencing of circulating-tumor DNA for tracking minimal residual disease in localized colon cancer. Ann Oncol (2019) 30(11):1804–12. doi: 10.1093/annonc/mdz390 31562764

[17] Reinert T Henriksen TV Christensen E Sharma S Salari R Sethi H. Analysis of plasma cell-free DNA by ultradeep sequencing in patients with stages I to III colorectal cancer. JAMA Oncol (2019) 5(8):1124–31. doi: 10.1001/jamaoncol.2019.0528 PMC6512280 31070691

[18] Chen G Peng J Xiao Q Wu HX Wu X Wang F. Postoperative circulating tumor DNA as markers of recurrence risk in stages II to III colorectal cancer. J Hematol Oncol (2021) 14(1):80. doi: 10.1186/s13045-021-01089-z 34001194 PMC8130394

[19] Parikh AR Van Seventer EE Siravegna G Hartwig AV Jaimovich A He Y. Minimal residual disease detection using a plasma-only circulating tumor DNA assay in patients with colorectal cancer. Clin Cancer Res (2021) 27(20):5586–94. doi: 10.1158/1078-0432.CCR-21-0410 PMC8530842 33926918

[20] Henriksen TV Tarazona N Frydendahl A Reinert T Gimeno-Valiente F Carbonell-Asins JA. Circulating tumor DNA in stage III colorectal cancer, beyond minimal residual disease detection, toward assessment of adjuvant therapy efficacy and clinical behavior of recurrences. Clin Cancer Res (2022) 28(3):507–17. doi: 10.1158/1078-0432.CCR-21-2404 PMC9401484 34625408

[21] Ogaard N Reinert T Henriksen TV Frydendahl A Aagaard E Orntoft MW. Tumour-agnostic circulating tumour DNA analysis for improved recurrence surveillance after resection of colorectal liver metastases: A prospective cohort study. Eur J Cancer (2022) 163:163–76. doi: 10.1016/j.ejca.2021.12.026 35074652

[22] Morris VK Yothers G Kopetz S Jacobs SA Lucas PC Iqbal A. NRG-GI005 (COBRA): Phase II/III study of circulating tumor DNA as a predictive biomarker in adjuvant chemotherapy in patients with stage II colon cancer. J Clin Oncol (2020) 38(4_suppl):TPS261–1. doi: 10.1200/JCO.2020.38.4_suppl.TPS261

[23] Taniguchi H Nakamura Y Kotani D Yukami H Mishima S Sawada K. CIRCULATE-Japan: Circulating tumor DNA-guided adaptive platform trials to refine adjuvant therapy for colorectal cancer. Cancer Sci (2021) 112(7):2915–20. doi: 10.1111/cas.14926 PMC8253296 33931919

[24] Tie J Cohen JD Lahouel K Lo SN Wang Y Kosmider S. Circulating tumor DNA analysis guiding adjuvant therapy in stage II colon cancer. N Engl J Med (2022) 386(24):2261–72. doi: 10.1056/NEJMoa2200075 PMC9701133 35657320

[25] Schraa SJ van Rooijen KL van der Kruijssen DEW Rubio Alarcon C Phallen J Sausen M. Circulating tumor DNA guided adjuvant chemotherapy in stage II colon cancer (MEDOCC-CrEATE): study protocol for a trial within a cohort study. BMC Cancer (2020) 20(1):790. doi: 10.1186/s12885-020-07252-y 32819390 PMC7441668

[26] Nors J Henriksen TV Gotschalck KA Juul T Sogaard J Iversen LH. IMPROVE-IT2: implementing noninvasive circulating tumor DNA analysis to optimize the operative and postoperative treatment for patients with colorectal cancer - intervention trial 2. study protocol. Acta Oncol (2020) 59(3):336–41. doi: 10.1080/0284186X.2019.1711170 31920137

[27] Lonardi S Montagut C Pietrantonio F Elez E Sartore-Bianchi A Tarazona N. The PEGASUS trial: Post-surgical liquid biopsy-guided treatment of stage III and high-risk stage II colon cancer patients. J Clin Oncol (2020) 38(15_suppl):TPS4124–TPS4124. doi: 10.1200/JCO.2020.38.15_suppl.TPS4124

[28] Gong J Hendifar A Gangi A Zaghiyan K Atkins K Nasseri Y. Clinical applications of minimal residual disease assessments by tumor-informed and tumor-uninformed circulating tumor DNA in colorectal cancer. Cancers (Basel) (2021) 13(18):4547. doi: 10.3390/cancers13184547 34572774 PMC8471730

[29] Chan HT Nagayama S Chin YM Otaki M Hayashi R Kiyotani K. Clinical significance of clonal hematopoiesis in the interpretation of blood liquid biopsy. Mol Oncol (2020) 14(8):1719–30. doi: 10.1002/1878-0261.12727 PMC7400786 32449983

[30] Chin YM Shibayama T Chan HT Otaki M Hara F Kobayashi T. Serial circulating tumor DNA monitoring of CDK4/6 inhibitors response in metastatic breast cancer. Cancer Sci (2022) 113(5):1808–20. doi: 10.1111/cas.15304 PMC9128178 35201661

[31] Chin YM Takahashi Y Chan HT Otaki M Fujishima M Shibayama T. Ultradeep targeted sequencing of circulating tumor DNA in plasma of early and advanced breast cancer. Cancer Sci (2021) 112(1):454–64. doi: 10.1111/cas.14697 PMC7780051 33075187

[32] Pittella-Silva F Chin YM Chan HT Nagayama S Miyauchi E Low SK. Plasma or serum: Which is preferable for mutation detection in liquid biopsy? Clin Chem (2020) 66(7):946–57. doi: 10.1093/clinchem/hvaa103 32516802

[33] Rose Brannon A Jayakumaran G Diosdado M Patel J Razumova A Hu Y. Enhanced specificity of clinical high-sensitivity tumor mutation profiling in cell-free DNA via paired normal sequencing using MSK-ACCESS. Nat Commun (2021) 12(1):3770. doi: 10.1038/s41467-021-24109-5 34145282 PMC8213710

[34] Hu Y Ulrich BC Supplee J Kuang Y Lizotte PH Feeney NB. False-positive plasma genotyping due to clonal hematopoiesis. Clin Cancer Res (2018) 24(18):4437–43. doi: 10.1158/1078-0432.CCR-18-0143 29567812

[35] Mayrhofer M De Laere B Whitington T Van Oyen P Ghysel C Ampe J. Cell-free DNA profiling of metastatic prostate cancer reveals microsatellite instability, structural rearrangements and clonal hematopoiesis. Genome Med (2018) 10(1):85. doi: 10.1186/s13073-018-0595-5 30458854 PMC6247769

[36] Razavi P Li BT Brown DN Jung B Hubbell E Shen R. High-intensity sequencing reveals the sources of plasma circulating cell-free DNA variants. Nat Med (2019) 25(12):1928–37. doi: 10.1038/s41591-019-0652-7 PMC7061455 31768066

[37] Bacon JVW Annala M Soleimani M Lavoie JM So A Gleave ME. Plasma circulating tumor DNA and clonal hematopoiesis in metastatic renal cell carcinoma. Clin Genitourin Cancer (2020) 18(4):322–31.e2. doi: 10.1016/j.clgc.2019.12.018 32046920

[38] Chan HT Chin YM Nakamura Y Low SK. Clonal hematopoiesis in liquid biopsy: From biological noise to valuable clinical implications. Cancers (Basel) (2020) 12(8):2277. doi: 10.3390/cancers12082277 32823942 PMC7463455

[39] Jin S Zhu D Shao F Chen S Guo Y Li K. Efficient detection and post-surgical monitoring of colon cancer with a multi-marker DNA methylation liquid biopsy. Proc Natl Acad Sci U.S.A. (2021) 118(5):e2017421118. doi: 10.1073/pnas.2017421118 33495330 PMC7865146

[40] Musher BL Melson JE Amato G Chan D Hill M Khan I. Evaluation of circulating tumor DNA for methylated BCAT1 and IKZF1 to detect recurrence of stage II/Stage III colorectal cancer (CRC). Cancer Epidemiol Biomarkers Prev (2020) 29(12):2702–9. doi: 10.1158/1055-9965.EPI-20-0574 32958500

[41] Marcuello M Vymetalkova V Neves RPL Duran-Sanchon S Vedeld HM Tham E. Circulating biomarkers for early detection and clinical management of colorectal cancer. Mol Aspects Med (2019) 69:107–22. doi: 10.1016/j.mam.2019.06.002 31189073

