# Supplementary material for: Tumor-informed or tumor-agnostic circulating tumor DNA as a biomarker for risk of recurrence in resected colorectal cancer patients
Source: Front Oncol. 2023 Jan 26;12:1055968. doi: 10.3389/fonc.2022.1055968 (PMC9909342; doi:10.3389/fonc.2022.1055968)
Supplement: Supplementary file 2 [file Image_1.pdf]

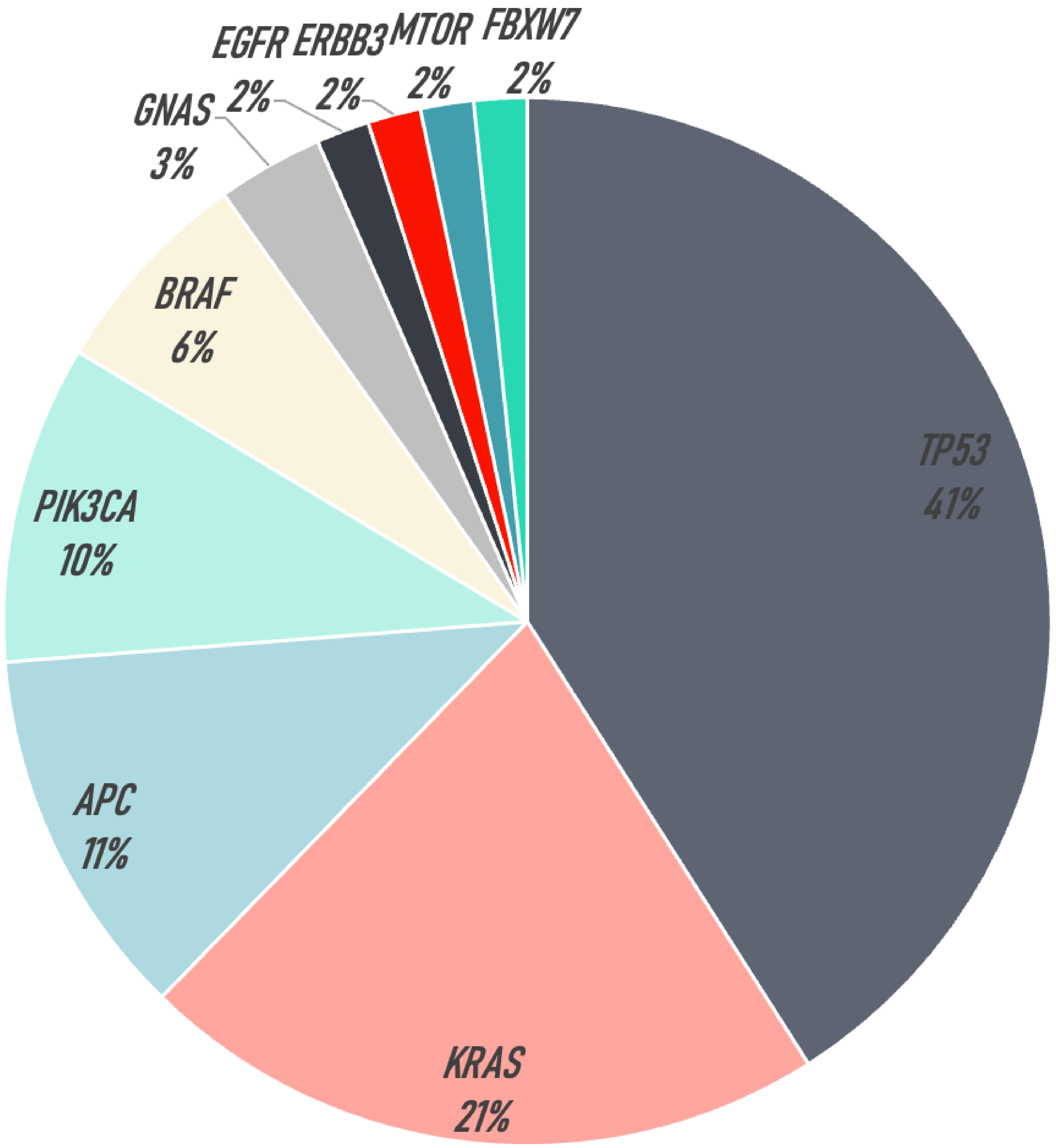

Figure S1. Distribution of genomic alterations detected from tumor tissues.

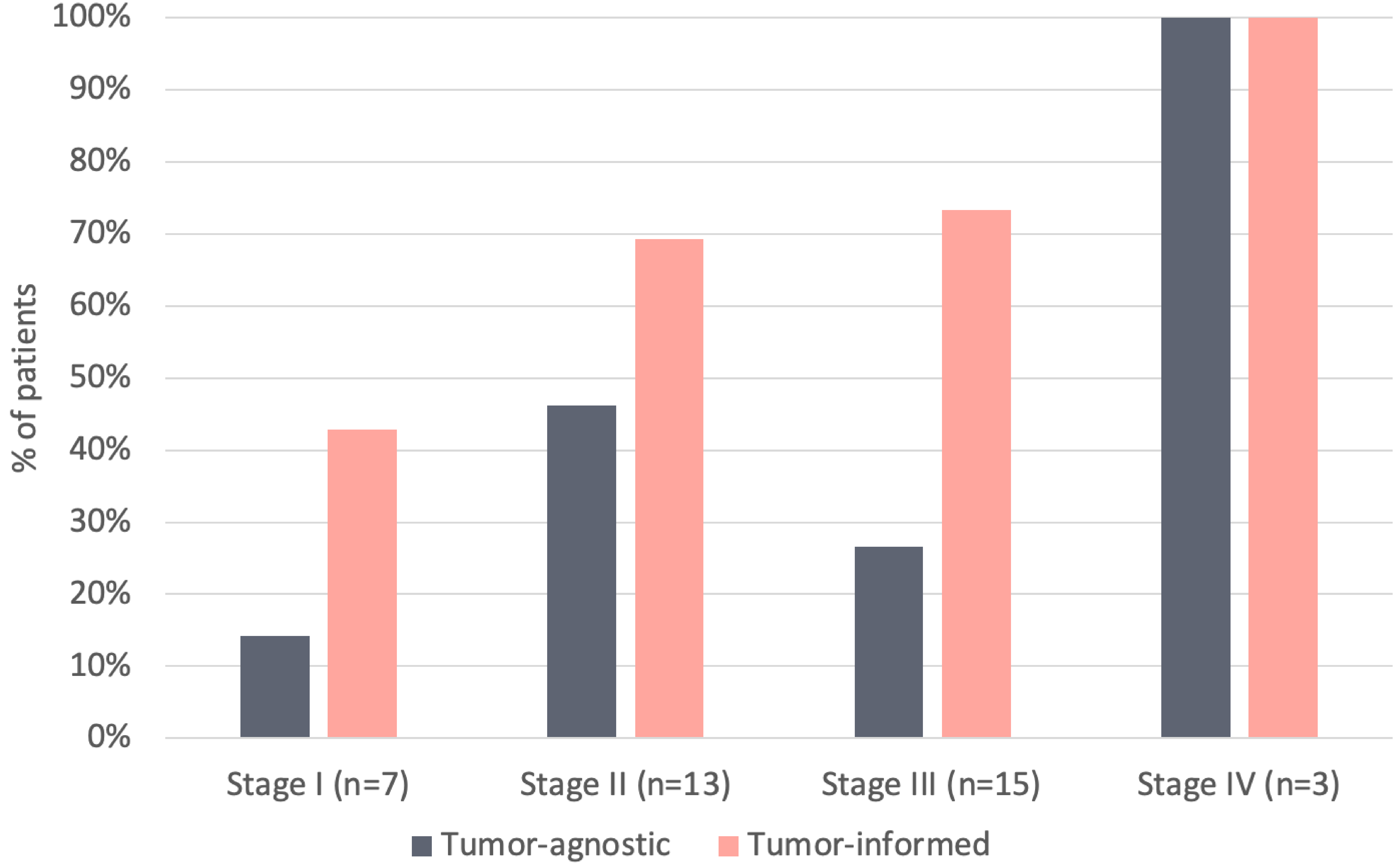

Figure S2. Pre-operatively ctDNA detection rate using tumor-informed and tumor-agnostic ctDNA testing by pathological stage.

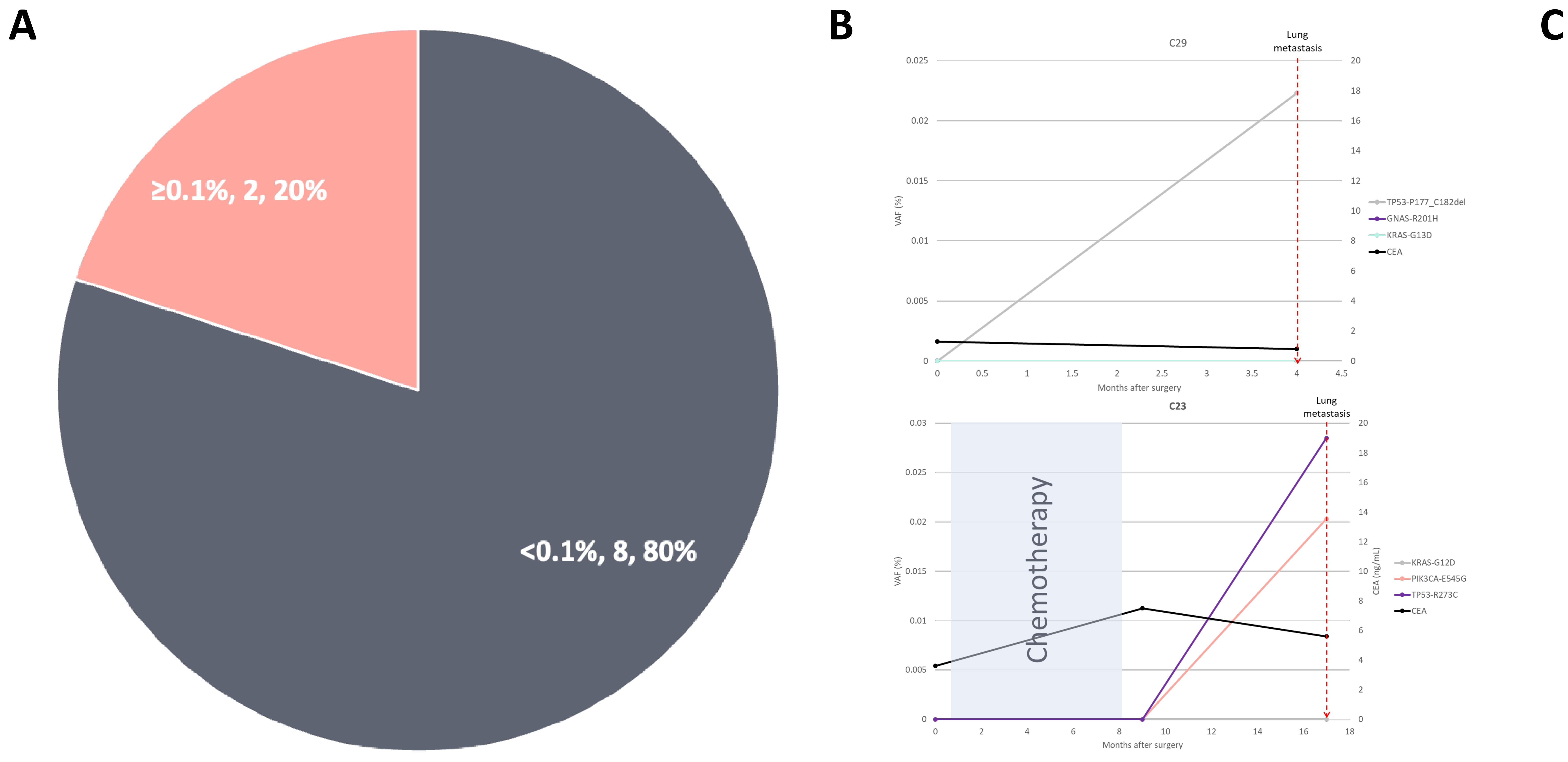

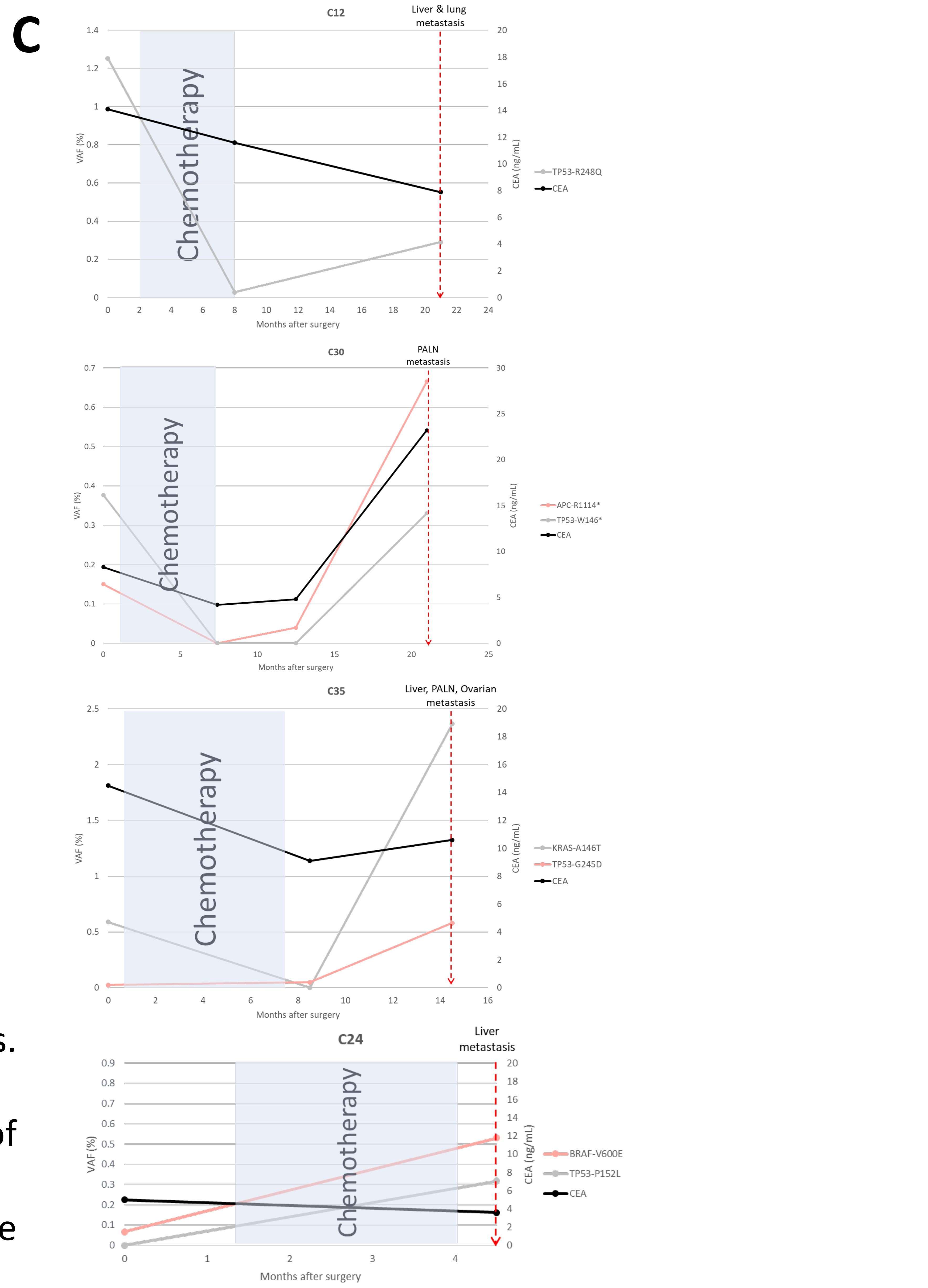

Figure S3. Recurrence detection sensitivity using tumor-informed and tumor-agnostic approaches

(A) Variant allele frequency (VAF) distribution of ctDNA alterations detected from serial monitoring samples. VAF < 0.1% is below tumor-agnostic detection threshold.

(B) Two patients who have developed recurrence with ctDNA alterations detected below the detection limit of the tumor-agnostic approach in serial monitoring samples.

(C) Four patients who have developed recurrence with alterations detected above the detection limit of the tumor-agnostic approach in serial monitoring samples.

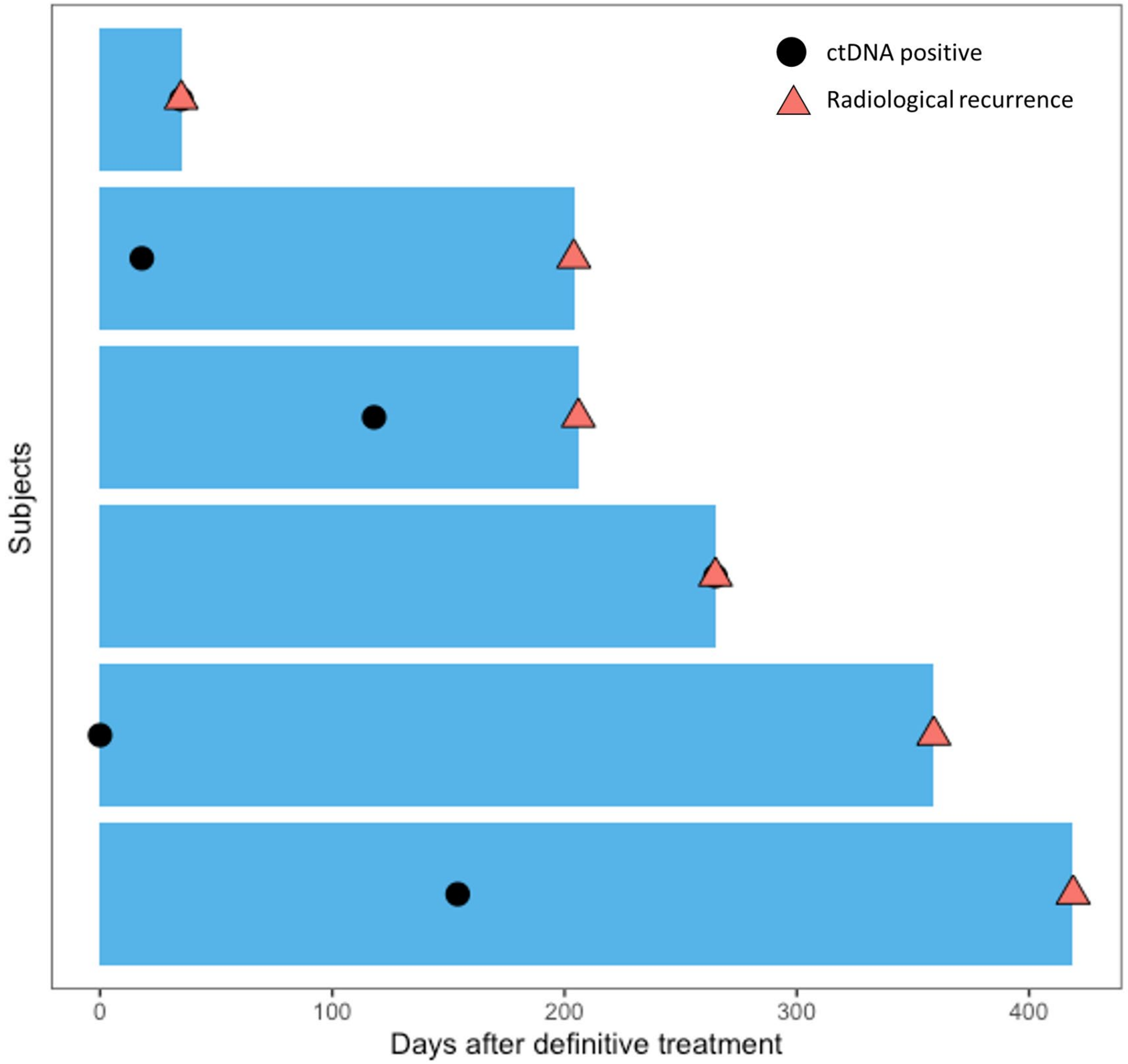

Figure S4. Swimmer plot of patients who have developed radiological recurrence and their post-definitive treatment ctDNA status.

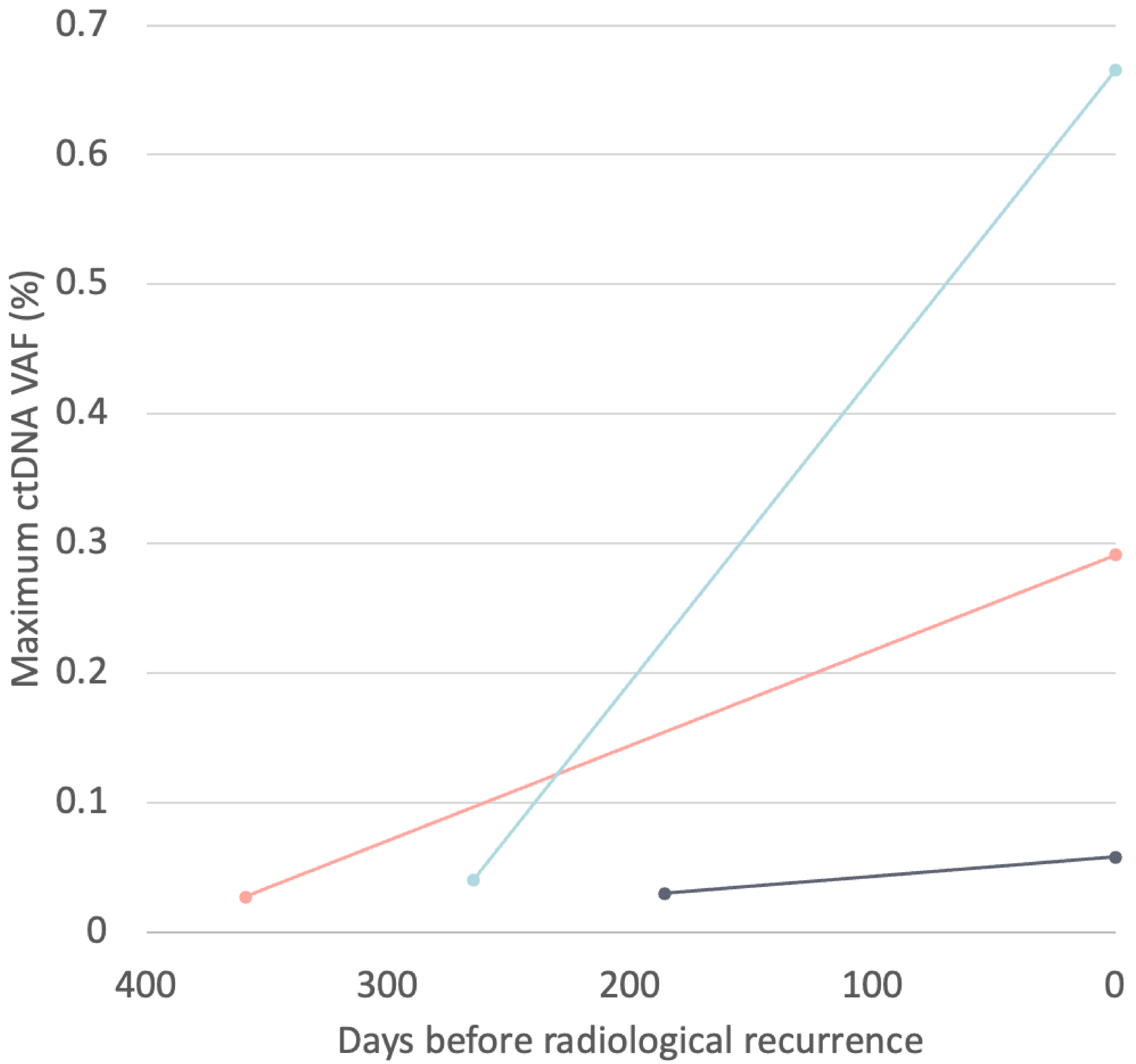

Figure S5. Increase in ctDNA VAF from ctDNA detection to radiological imaging
A median of 10 fold increase in ctDNA VAF was observed from ctDNA detection to radiologic detection in three patients who have developed recurrence. Timepoints prior to completion of definitive treatment was omitted. Each colored curve represents data from a different patient.
